# Prevalence and factors associated with overweight and obesity among private kindergarten school children in Bahirdar Town, Northwest Ethiopia: cross-sectional study

**DOI:** 10.1186/s13104-016-2308-8

**Published:** 2017-01-04

**Authors:** Yoseph Tadesse, Terefe Derso, Kefyalew Addis Alene, Molla Mesele Wassie

**Affiliations:** 1 Department of Human Nutrition, Institute of Public Health, College of Medicine and Health Sciences, University of Gondar, Gondar, Ethiopia; 2 Department of Epidemiology and Biostatistics, Institute of Public Health, College of Medicine and Health Sciences, University of Gondar, Gondar, Ethiopia

**Keywords:** Overweight, Obesity, Kindergarten, Malnutrition, Children, Ethiopia

## Abstract

**Background:**

In Sub-Saharan Africa, most nutrition efforts have concentrated on under-nutrition in children. However, national surveys rarely report the high prevalence of overweight and obesity among children. Likewise, in Ethiopia there is growing recognition of the emergence of a “double-burden” of malnutrition, with under and over nutrition occurring simultaneously among children, especially allied with improvements in socio-economic conditions. Hence, the study aimed to assess the prevalence and factors associated with overweight and obesity among private kindergarten school children aged 3–6 years in Bahirdar town, Northwest Ethiopia.

**Methods:**

A school-based cross sectional study was conducted in Bahirdar Town, northwest Ethiopia from August to September, 2015. Anthropometric measurements such as weight and height were taken from 462 private Kindergarten preschool children aged 3–6 years; socio-economic and demographic factors and feeding practices were collected by interviewing the, mothers or caregivers of the children. The z-score values for BMI-for-age of children were generated using Emergency Nutrition Assessment (ENA) for Standardized Monitoring and Assessment of Relief Transitions (SMART) 2011. Binary logistic regression model was used to identify factors associated with overweight and obesity in children. Odds ratio with 95% confidence interval (CI) was calculated to show the strength of association.

**Results:**

The overall prevalence of overweight and obesity was 6.9% [95% CI 2.4, 11.4]. The prevalence of overweight and obesity were 4.1 and 2.8%, respectively. The odds of overweight and obesity was higher among children with high dietary diversity score (DDS) [AOR = 5.12, 95% CI 1.42, 18.47], family size of less than five [AOR = 4.76, 95% CI 1.84, 12.31] and a family having a private car [AOR = 3.43, 95% CI 1.02, 11.49].

**Conclusions:**

The prevalence of overweight and obesity among private kindergarten preschool children in the study area was high. Interventions on improving feeding practice and doing physical activities are important for the control of overweight and obesity among children in urban settings.

## Background

Overweight and obesity among children are defined as Body Mass Index (BMI)-for-age (BMI-for-age) >2 but ≤3 standard deviations (SD) and >3 (+3 SD) standard deviations (+3 SD), respectively [1]. Childhood obesity leads to the risk of obesity in adulthood and long-term health consequences such as type II diabetes, cardiovascular disease (CVD), hypertension, hyperlipidemia, certain forms of cancer, as well as respiratory and skin problems [2]. Moreover, obese people, particularly children, often have low self-esteem, poor school performance and social interaction [2, 3]. It is one of the most serious public health challenges of the twenty-first century [3]. Globally, in 2010 the number of preschool children suffering from overweight and obesity was estimated to be over 43 million, 81% of these cases were living in developing countries [4]. As a result, in developing countries there is a growing recognition of the emergence of a “double burden” of malnutrition, with under and over nutrition occurring simultaneously among children, particularly allied with improvement of economic conditions [5]. However, in sub-Saharan Africa including Ethiopia, most nutrition efforts have still concentrated on under-nutrition in children [3, 6, 7].

Studies in developing countries have shown that the prevalence of overweight and obesity among children are increasing overtime and it varies from country to country 20.1% in Kenya [8], 21.1% in urban Vietnam [9], 23.6% in Nigeria [10], 9% in the Recife Metropolitan Region [11] and 8.42% in Punjab India [12].

According to the Ethiopian demography and health survey (EDHS) 2014 mini report, the prevalence of obesity among children under five years of age was 5% in Benishangul Gumuz and 6% in Addis Ababa [13]. The combined prevalence of overweight and obesity among children aged 3–6 years was 10.7% in southern Ethiopia [14].

Previous studies conducted in various setting identified several risk factors for overweight and obesity among children, including: socio-economic status of the family [15], family size [8, 16], educational status of mothers [17], physical activities [8, 14, 18], dietary habit and a family history of overweight and obesity [19]. Previous researches were conducted among children were more focused on under nutrition than over nutrition. There are few studies conducted on obesity and overweight, particularly in Ethiopia, and none of them included private kindergarten (KG) school children aged 3–6 years in urban settings that was investigated in our study. Thus, the study aimed to assess prevalence and associated factors of overweight and obesity among private kindergarten preschool children in Bahirdar town, Northwest Ethiopia.

## Methods

School based cross-sectional study was conducted from August to September, 2015 among private kindergarten school children aged 3–6 years in Bahirdar town.

Bahirdar town is the capital city of Amhara regional state in the Northwest part of Ethiopia. It is found 564 km far from Addis Ababa, the capital of Ethiopia. The town has an estimated population size of 256,999 [20]. It has also 31 private Kindergarten schools. According to the information obtained from the Bahirdar town education office 6646 KG students are attending their private KG education.

All school children aged 3–6 years attending private Kindergarten (KG) schools were included in the study. Sample size was calculated using Epi-info version 7 by considering the following assumptions; 18% prevalence of overweight and obesity taken from Southern Ethiopia study [14], 95% level of confidence, 5% margin of error, 5% non-response rate, and a design effect of 2. A minimum sample size of 476 was obtained, and multi stage sampling techniques was used to select the study participants. Out of a total of 31 KG schools found in Bahirdar town 7 were selected using simple random sampling technique. Then, the total number of KG children was proportionally allocated for each KG (1–7). Finally, children from each KG were selected using a lottery method.

The dependent variable of the study (i.e. overweight and obesity) was assessed based on the WHO recommendation [21]. Overweight was defined as children more than two standard deviations (+2 SD) but ≥3 standard deviation above the median body mass index (BMI) for age (BMI-for-age). Obesity was defined as children more than three standard deviations (+3 SD) above the median BMI-for-age [1]. The combined prevalence of overweight and obesity was determined by the sum of specific prevalence of overweight and obesity.

Independent variables included in the study were: socio-economic and demographic characteristics of parents and children (age of children, sex of children, marital status of mother/caregivers, religion of the mother/caregivers, family size, monthly income, occupation and educational status of parents), feeding practice(frequency of snack and dietary diversity score/DDS/). Dietary diversity was assessed by based on the WHO eight food grouping: grains, roots and tubers; legumes, nuts and seeds; dairy products; flesh foods; eggs; vitamin A-rich fruits and vegetables; other fruits and vegetables; and any foods made with oil, fat, or butter [21]. The dietary diversity score (DDS) was rank into three sub groups (tertiles):if the child consumed: 0 to 2 food groups classified as “poor”, 3 to 5 food groups classified as “medium”, six and above food groups classified as “high” in the previous day preceding the survey [21].

Data from the mothers or caregivers of the children were collected in home to home visits using structured, pretested, and interviewer administered questionnaire to obtain socio- economic and demographic variables as well as feeding practice of the mothers. To maintain its consistency, the questionnaire was first translated from English to Amharic, the native language of the study area, and was retranslated back to English by professional translators and Public Health experts. Weight and height of the child were measured using standardized and calibrated equipments at the kindergarten school [23]. Weight of children was measured using beam balance with light closing, and was measured to the nearest 0.1 kg, and height of children was measured to the nearest 0.1 cm on standing position without shoes [22].

Eight nurses were participating in the data collection process after getting a two days intensive training on the objective of the study, confidentiality of information, and anthropometric measurement. All filled questioners were checked daily for completeness, accuracy and consistency by the supervisor and the primary investigator.

Data were checked for completeness and were entered into Epi-info version 7. Data were then exported to Statistical Package for Social science (SPSS) version 20 for analysis. The z-score values for BMI-for-age (BAZ) of children were generated using ENA for SMART 2011. Frequencies and cross tabulations were used to summarize descriptive statistics, tables and graphs were used for data presentation. Binary logistic regression models were used to identify variables which have an association with the dependent variable. Variables found to have p value up to 0.2 in the bivariate analysis, entered into multivariate logistic regressions for controlling the possible effect of confounders and finally the variables which have significant association were identified on the basis of OR, with 95% CI.

### Ethical considerations

Ethical clearance was obtained from the institutional review boards of the University of Gondar. Permission was obtained from Amhara Regional Health Bureau, Bahirdar Town education office and the selected schools. Informed consent was obtained from the mother or caregiver of each child after providing information about the purpose of the study. In order to keep confidentiality of any information provided by study participants, the data collection procedure were anonymous.

## Results

A total of 462 kindergarten preschool children-pairs with mothers/caregivers (with a response rate of 97%) participated in the study. The majority of mothers/caregivers were married (92.2%) and Orthodox Christian (84.6%). Less than half of mothers/caregivers were housewives (42.2%) and completed secondary school (46.5%). Half (51.1%) of the children were females. The mean age (± SD) of the children was 54.91 (±11.65) months (Table 1).

**Table 1:** Socio-economic and demographic characteristics of mothers with KG school children aged 3–6 years in Bahirdar town, Northwest Ethiopia, 2015

Characteristics	Frequency	Percent (%)
Age of child (in months)		
36–60 months	331	71.6
61–72 months	131	28.4
Sex of child		
Female	236	51.1
Male	226	48.9
Grade level of the child		
KG-1	181	39.2
KG-2	150	32.5
KG-3	131	28.4
Marital status of mothers		
Married	426	92.2
Others^a^	36	7.8
Religion		
Orthodox Christians	391	84.6
Muslim	40	8.7
Others^b^	31	6.7
Educational status of mothers		
Unable to write and read	27	5.8
Able to write and read	26	5.6
Primary education	147	31.8
Secondary	215	46.5
Above secondary school	47	10.2
Occupational status of mothers		
Housewife	195	42.2
Government employee	124	26.8
Merchant	77	16.7
Non Governmental Organization (NGO) employee	36	7.8
Others^c^	30	6.5
Family size		
<5	245	53
≥5	217	47
Household monthly income in Ethiopia Birr		
<2000	170	36.8
≥2000	292	63.2
Family/private car for child transport		
Yes	30	6.5
No	432	93.5

^a^Single, divorced and widowed

^b^Protestant and Catholic

^c^Private workers, daily laborer

### Dietary diversity of children

**Fig. 1:**
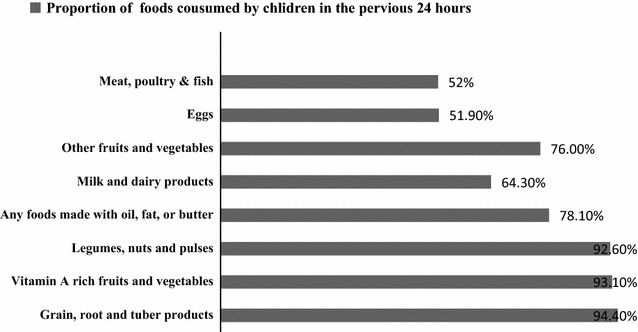
Dietary diversity of KG school children aged 3–6 years in the last 24 h preceding the date of survey in Bahirdar Town, of Northwest Ethiopia, 2015

Vast majority of children consumed grain, root and tuber products (94.4%), vitamin-A rich fruits and vegetables (93.1%), and any foods made with oil, fat, or butter(78.1%) in the previous 24-h (Fig. 1).

### Prevalence of overweight and obesity among KG children

**Fig. 2:**
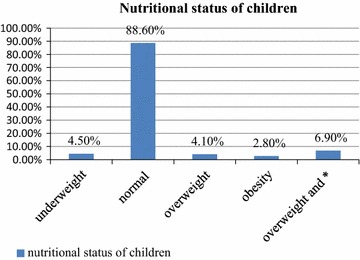
Status of Kindergarten childhood weight in Bahirdar Town, Northwest Ethiopia. Factors associated with overweight and obesity among KG children. * obesity

The combined prevalence of overweight and obesity was 6.9% [95% CI 2.4, 11.4]. The prevalence of overweight and obesity in the study participants were 4.1% [95% CI 0.6, 7.6] and 2.8% [95% CI 0.1, 5.1], respectively (Fig. 2).

In bivariate analysis age and sex of the child, dietary diversity score (DDS), frequency of snack, family size and family/private car were found with a p-value of less than 0.2. However, The result of multivariate analysis revealed that dietary diversity score (DDS), family size and family/private car go to school and back to home were independently and significantly associated with overweight and obesity. Accordingly, the odds of overweight and obesity was higher among children with high dietary diversity score (DDS) [AOR = 5.12, 95% CI 1.42, 18.47] compare to poor dietary diversity score. The study also found that a family size less than five [AOR = 4.76, 95% CI 1.84, 12.31] was associated with increased risk of overweight and obesity among children compared to family size of greater than five. Another risk factor was family/private car, children who were transported by family car to school and back to home more likely to overweight and obesity [AOR = 3.43, 95% CI 1.02, 11.49] compared to the counter parts. Hosmer and Lemeshow test indicate excellent fit, that was p value greater than 0.5 (Table 2).

**Table 2:** Factors associated with overweight and obesity among KG children in Bahirdar Town, Northwest Ethiopia, 2015

Variables	Overweight/obesity		Crude odds ratio (95% CI)	Adjusted odds ratio (95% CI)
	Yes (n#)	No (n#)		
DDS				
Low	3	110	1.00	1
Medium	6	157	1.40 (0.34, 6.72)	1.35 (0.31, 5.75)
High	23	163	5.17 (1.51–17.65)	5.12 (1.42–18.47)*
Frequency of snack				
None	4	80	1	1
One	13	255	1.02(0.32, 3.21)	2.07 (0.58, 7.31)
Two	15	95	3.15(1.00–9.89	0.68 (0.19–2.39)
Family/private care				
No	27	405	1	1
yes	5	25	3.00 (1.06, 8.45)	3.43 (1.02–11.49)*
Family size				
<5	25	219	3.44 (1.45, 8.12)	4.76 (1.84–12.31)*
≥5	7	211	1	1
Age of child				
36–60 months	19	312	1	
61–72 months	13	118	1.81 (0.87, 3.78)	

* Significant at p value < 0.05

## Discussion

This study revealed that, the combined prevalence of overweight and obesity was 6.9% [95% CI 2.4, 11.4], which was similar with other findings in Ethiopia 5 and 10.7% [13, 14], 8.5% in Africa [7], 8.42% in Punjab India [12] and the global prevalence of overweight and obesity was 7% [19].

However, the current study was lower than study reports from different countries; 20.1% in Kenya [8], 23.6% in Nigeria [10] and 21.1% in urban Vietnam [9]. The observed discrepancy might be due to socio-cultural variations like high socio-economic status in the previous studies. This may led to changes in lifestyle such as the introduction of negative eating habits and increased sedentary behavior.

In this study, the odd of overweight and obesity was higher among children who had high dietary diversity score compared to children with low dietary diversity score. This finding was supported with another study report in southern Ethiopia [14] and dietary diversity might be a determinant factor for the dual existent of under and over nutrition [23]. The possible reason could be an increased intake of high-energy dense foods as the dietary diversity score increases which will have a significant influence on weight gain of children.

This study revealed that family size less than five was associated with increased risk of overweight and obesity among children compared to family size larger than five. Studies conducted in Kenya [8] had similar results. A smaller family size might imply less sharing of available food and other family resources and allows families to tender better nutrition, which in a tremendous state of affairs may well contribute to excessive energy intake and obesity.

In the present study, Children who go to school and back to home with a private/family car were 3.43 times more likely to be overweight/obese as compared to the family who had no family/private car. This finding was congruent with other findings [8, 18]. It could be that a family/private car is not only an indicator of a sedentary life status but also an ideal indicator for identifying family socio economic status. A number of studies support the finding that SES is significantly associated with childhood overweight/obesity.

### Limitations of this study

Despite the data was collected by experienced and trained data collectors and supervisors, recall bias and social desirability bias by participants on variables like the dietary habits(dietary diversity) might be happened. Besides; BMI fails to distinguish between fat and fat-free mass (muscle and bone), also waist circumference is not addressed in the study. Finally, other factors which can affect excess body weight like sedentary and behaviors physical activity, genetic factor, health condition and drug use of participants were not addressed in this study.

## Conclusions

The finding of this study revealed that a significant number of children were inclined to overweight and obesity. Nutrition education on feeding practices and physical activities should be boosted. Moreover, further study is recommended to explore other potential factors associated with overweight/obesity that were not included in the present study.

## Abbreviations

AOR: adjusted odds ratio
COR: crude odds ratio
DDS: dietary diversity score
KG: kindergarten
BMI: body mass index
SPSS: statistical program for social sciences
